# Biologically Inspired Deep Learning Model for Efficient Foveal-Peripheral Vision

**DOI:** 10.3389/fncom.2021.746204

**Published:** 2021-11-22

**Authors:** Hristofor Lukanov, Peter König, Gordon Pipa

**Affiliations:** ^1^Department of Neuroinformatics, Institute of Cognitive Science, Osnabrück University, Osnabrück, Germany; ^2^Department of Neurobiopsychology, Institute of Cognitive Science, Osnabrück University, Osnabrück, Germany; ^3^Department of Neurophysiology and Pathophysiology, Center of Experimental Medicine, University Medical Center Hamburg-Eppendorf, Hamburg, Germany

**Keywords:** space-variant vision, active vision, foveal vision, peripheral vision, deep learning-artificial neural network (DL-ANN), bottom-up attention, top-down attention

## Abstract

While abundant in biology, foveated vision is nearly absent from computational models and especially deep learning architectures. Despite considerable hardware improvements, training deep neural networks still presents a challenge and constraints complexity of models. Here we propose an end-to-end neural model for foveal-peripheral vision, inspired by retino-cortical mapping in primates and humans. Our model has an efficient sampling technique for compressing the visual signal such that a small portion of the scene is perceived in high resolution while a large field of view is maintained in low resolution. An attention mechanism for performing “eye-movements” assists the agent in collecting detailed information incrementally from the observed scene. Our model achieves comparable results to a similar neural architecture trained on full-resolution data for image classification and outperforms it at video classification tasks. At the same time, because of the smaller size of its input, it can reduce computational effort tenfold and uses several times less memory. Moreover, we present an easy to implement bottom-up and top-down attention mechanism which relies on task-relevant features and is therefore a convenient byproduct of the main architecture. Apart from its computational efficiency, the presented work provides means for exploring active vision for agent training in simulated environments and anthropomorphic robotics.

## 1. Introduction

The biological visual system has served as a template and inspiration in Computer Vision in many ways. Improvements in hardware allowed Deep Learning and specifically Convolutional Neural Networks (CNNs) to gain a lot of interest from the research community, which now dominate vision models in this field (O'Mahony et al., 2019). The layered structure and information processing mechanisms, that CNNs rely on, resemble more closely biological systems than previously used machine learning approaches. This research has achieved human and even superhuman performance in many benchmarks, such as hand-written digit recognition, image classification and so on Ciresan et al. (2012) and Chen et al. (2015).

Despite the success story of modelling the biological visual system by deep neural networks, there are several qualitative differences in properties as well as in performance. Such differences can be found in the learning architecture, with feed-forward Artificial Neural Networks (ANNs) dominating the field of Deep Learning, as well as the homogeneous resolution and rectangular shape of the visual data used for training, in contrast to the space-variant sampling in the human retina. Despite the improvement of performance of ANNs over classical Computer Vision, biological vision retains dominance in many visual problems such as object detection and classification with object occlusions, noisy data and in cluttered scenes (Dodge and Karam, 2017; Geirhos et al., 2018). Additionally, the good performance of ANNs comes at a significant computational cost, that requires dedicated hardware for training and inference. Most current image and video datasets reflect these limitations and provide only low-resolution images, as training in high resolution is computationally challenging. This complicates applications of object classification at a distance, essential for processing security camera footage, traffic control and so on. Deep architectures further complicate this issue as video memory limitations of GPU units quickly render training impossible. In summary, despite many similarities, there are major discrepancies between the biological template and ANNs used to model function and performance.

Of specific interest for the present study are the space-variant sampling by the human retina paired with the mechanisms of generating sequences of fixations and the ability to focus processing in a highly energy efficient manner. The fovea, a small region on the retina around 1.5 mm in diameter (Polyak, 1941; Riordan-Eva et al., 2011), densely packed with cone photoreceptors, allows for perceiving visual details with high acuity. It accounts for a relatively small portion of about 1° of eccentricity from the center of the field of view. The neighboring parafoveal region, still rich in cone cells, extends to about 4–5° and provides slightly lower spatial resolution. Together, fovea and parafovea, lie in the macula lutea, and contribute to what is commonly referred to as central vision. Beyond the macula, a rapid decrease in cone cell density is observed, responsible for the low acuity in our peripheral vision. By sampling the visual signal in a space-variant manner, instead of uniformly, a balanced solution allows the ventral stream in the visual cortex to only process a small area in high resolution while retaining a large field of view with lower spatial resolution in the periphery (Daniel and Whitteridge, 1961; Cowey and Rolls, 1974). Furthermore, such sampling is cost efficient with respect to the ability to retain sufficient detail while reducing processing requirements. It has been estimated that processing costs for the brain are reduced by a factor of 350 times less than a hypothetical full-resolution visual signal received at the human retina (Weber and Triesch, 2009). While the high-resolution part of vision covers only a small portion of the perceived scene, saccadic eye-movements, driven by visual attention, allow for incremental collection of additional detail information. Thus, given the mostly static nature of our world and a short-term memory mechanism, space-variant vision compensates for the lack of uniform high acuity across the field of view. In summary, the limited spatial resolution at higher eccentricity might not be a bug, but a feature allowing focused energy efficient sampling of visual information.

Here, we propose an end-to-end deep neural network model of the ventral visual stream, inspired by the sampling mechanisms of the primate retina and the cortical magnification effect (Daniel and Whitteridge, 1961; Cowey and Rolls, 1974) in the visual cortex. Our method demonstrates a significant reduction in processing and memory costs due to the drastically smaller size of the neural network input and can be used for efficient object recognition and object tracking by an agent. We show that despite sampling only a 10^th^ of the image pixels, with a single saccade the model provides comparable classification accuracy and even outperforms a full-resolution network on video classification where it can exploit the sequential information, thus overcoming object occlusions or saccades to a wrong location. Furthermore, in order to efficiently guide eye movements, we propose an easy to implement attention mechanism based on feature saliency that can be used in a bottom-up manner to detect salient objects or top-down for locating or tracking a specific object by the agent. We believe that this research can assist advancements of active vision in anthropomorphic robotics and agent training in simulated environments.

## 2. Related Work

### 2.1. Retinal Circuitry

Retinal Ganglion Cells (RGC) represent the last layer of neuronal cells in the retina that transmits the visual signal through the optic nerve and to the brain. Various types of RGCs have been identified in the retina (Field and Chichilnisky, 2007; Petrusca et al., 2007). Most prominently they can be classified into Parasol and midget cells. Parasol cells have larger receptive field (RF) size and have a greater presence in the periphery of the retina (Dacey and Petersen, 1992). They are specialized for low spatial resolution, motion detection and participate in achromatic vision (Livingstone and Hubel, 1988; Croner and Kaplan, 1995). In comparison, midget cells are characterized by smaller RFs than Parasol cells and have greater concentration in the fovea, where they receive input from just a single cone cell (Dacey and Petersen, 1992). They are specialized for high spatial acuity and color vision (Livingstone and Hubel, 1988). The dendritic tree diameter of both RGCs increase with eccentricity (Dacey and Petersen, 1992), intensifying the information compression even further than the distribution of photoreceptors. Thus, the signal from the ganglion cell layer, foveated due to distribution and RF size, represents the sole output of the retina and is of particular interest to the purposes of this paper.

### 2.2. Computational Methods for Foveation

Foveated sampling in the retina and the cortical magnification effect in the visual cortex are largely ignored in most computational models. However, there are a few exceptions that explore foveation techniques for vision models. One such method is the Exponential Cartesian Geometry (ECG) (Bandera and Scott, 1989; Scott and Bandera, 1990), proposed by Bandera and Scott. It serves the function of a foveation mechanism and is based on the principle of image pyramids. A central crop is made from the original image that retains its original high resolution and serves as the fovea. Then a number of rings are defined, surrounding the fovea—each one in a lower resolution achieved by downsampling the ring portion of the image to a lower resolution. This family of methods have successfully been used for object detection (Camacho et al., 1996; Arrebola et al., 1997, 1998), object tracking (Gomes et al., 2010) and segmentation (Camacho et al., 2003). Gomes et al. use ECG to perform fast parallel feature extraction (Gomes et al., 2010) and then perform object tracking using an ANN. The image pyramid structure of ECGs, however, have some downsides. They require specialized pyramid image algorithms in order to process the multiple scaled images. Although there are some implementations of such algorithms for ANNs (He et al., 2015; Fu et al., 2019; Pang et al., 2019), they introduce additional complexity and place constraints on the network architecture, which can be used only for specific tasks. Furthermore, working on multiple, separate image inputs creates additional problems for object recognition if an object is not located in a single ring but is split among two or more rings.

To avoid the complexity of working with multiple image scales, ECGs can be simplified by only using images in two resolutions – a uniformly downsampled image representing peripheral vision and a small high-resolution crop representing central vision. Such a model has been proposed by Karpathy et al. (2014) for large-scale video classification. This model has two streams—a high-resolution crop taken from the center of the image and a low-resolution downsampled version of the original image. The crop is used for classification, while the peripheral stream provides context. This approach takes advantage of the photographer's bias (Tseng et al., 2009)—an assumption that in human produced videos the region of interest is primarily located near the center of the image. This, however, is only a viable method when processing human made video signal and cannot be used for general purpose vision. Zhang et al. (2019) and Xia et al. (2020) propose an alternative that uses an attention mechanism to determine the location of the crop. First, the downsampled image is used by a CNN that produces a saliency map. This saliency map is then used in order to determine the location at which the high-resolution crop is taken. The crop, in turn, is used by another CNN that processes fine details. Although these models do not truly foveate the visual signal, they have good performance but have the disadvantage that at least two CNNs need to be trained separately. The same is valid for the forward pass of the model—it happens in several operations, some of which are non-differentiable and require separate networks. In comparison, our model is trained and tested in a single forward pass that processes central, peripheral information and determines location for the next saccade.

The models described so far used multi-resolution images to functionally approximate the principles of biological vision. Several models take a step toward biologically more plausible foveation technique. This is accomplished by using a single image and applying Gaussian blur to remove high spatial frequencies from its periphery (Almeida et al., 2017; Meĺıcio et al., 2018; Deza and Konkle, 2020). Almeida et al. (2017) demonstrate that foveation does not hinder object localization and classification performance over a certain size of the non-blurred fovea; and Deza and Konkle conclude that the blurring technique leads to a better generalization for scene recognition (Deza and Konkle, 2020). The main drawback of these models, however, is that even though they remove high frequencies from the periphery, the number of pixels in the foveated image remains the same as the original. There is no compression of the signal and therefore no decrease of computation costs.

A well-known method that mimics the retinal sampling more closely and additionally compresses the signal is the log-polar mapping (Daniel and Whitteridge, 1961; Schwartz, 1977, 1984). Sampling from a conventional image with coordinates *x*,*y* maps to a rectangular plane in the log-polar domain with coordinates ρ (eccentricity) and θ (angle). This type of mapping emulates the distribution of RGC cells in the retina and reduces the number of pixels in the transform. The resulting rectangular shape is also suitable for use with ANNs and because of the nature of the transformation, under certain conditions, is invariant to scale and rotation (Schwartz, 1984). For these reasons, the log-polar transform is a very popular foveation technique in many vision models (Wallace et al., 1994; Colombo et al., 1996; Kanan, 2013; Aboudib et al., 2016; Akbas and Eckstein, 2017; Ozimek et al., 2019; Daucé et al., 2020), as well as for specific tasks such as image registration (Wolberg and Zokai, 2000; Sarvaiya et al., 2009) and object detection and tracking (Jurie, 1999; Metta et al., 2004). Due to its biological plausibility and well-established place in similar vision models, we use this method as a baseline for performance of our model. While log-polar sampling (**Figures 2A,B**) accurately represents sampling in the retina, however, the log-polar transform produces severe spatial deformations and discontinuities (**Figure 2C**) that create difficulties for object recognition, as we later show in 4.

Foveal Cartesian Geometry (FCG) (Mart́ınez and Robles, 2006) was introduced as an alternative to log-polar transform with a highlight on avoiding some of the disadvantages present in previously mentioned computational methods in this chapter. It approximates the log-polar sampling by using a pseudo log-polar grid (Averbuch et al., 2001) instead. The difference is that pixels are sampled from concentric squares around the fovea (**Figure 2D**), instead of circles. The result can then perfectly fit into a rectangular shape with no gaps and only slight deformations on the diagonals (**Figure 2F**). Thus, FCG reduces the size of the foveated image, emulating retinal sampling, and preserves spatial relations between sampled pixels. In contrast to log-polar transform, this technique is also linear. FCG has been used for object tracking and detection (Mart́ınez and Robles, 2006), as well as in a cortical magnification model (Aboudib et al., 2015), although none of them use ANNs. Despite some indications that Cartesian foveation performs better with CNNs than log-polar mapping (Torabian et al., 2020), not much research has been done on the topic and we believe it to be a fruitful one for computational vision.

Thus, most described methods have one or more drawbacks. Primarily, space-variant sampling presents difficulties for providing a suitable input for ANNs, which are the state-of-the-art method for information processing in Computer Vision. Such is the case with log-polar sampling, as well as the ECG and multi-resolution methods that require separate streams for low- and high-resolution information that need to be integrated. Another property, pivotal for retinal sampling, is the proper distribution of processing power to accompany information reduction. For example, the Gaussian blur method removes high spatial frequencies at high eccentricity without reducing the number of pixels in the image. As a result, the same amount of processing power is designated equally to regions of fine and coarse detail. Lastly, log-polar transform and FCG exhibit some spatial deformations but do not have the other two drawbacks. These two methods are best suited for a deep learning model and therefore a main subject of the present study. As spatial deformations are greater in the log-polar transform technique, we use it as a baseline sampling method and base our model on FCG. We compare both techniques empirically in 4.

## 3. Methods

### 3.1. Model

**Figure 1:**
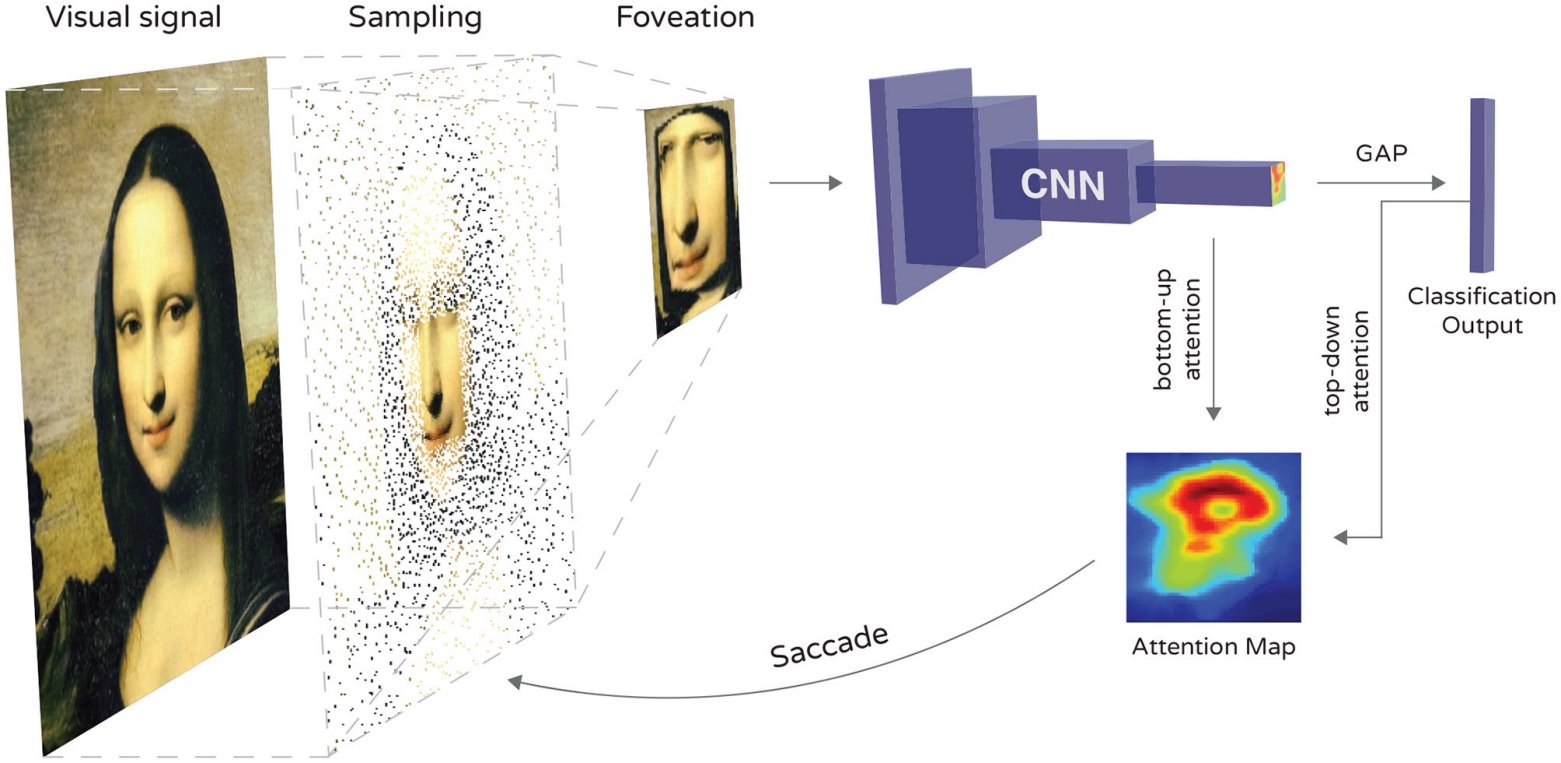
Diagram of the proposed model. The raw image input is foveated with FCG and classified by the CNN. Attention map is extracted from the last convolutional layer and used for bottom-up attention utilizing salient features from the observation. A Global Average Pooling (GAP) layer is used before the classification output layer. Its usage assists the top-down attention mechanism in augmenting the attention map such that features specific to certain class(es) of objects are inhibited or prioritized. The maximum intensity in the attention map is then used to determine the location to which the fovea should move in the next time step.

**Figure 2:**
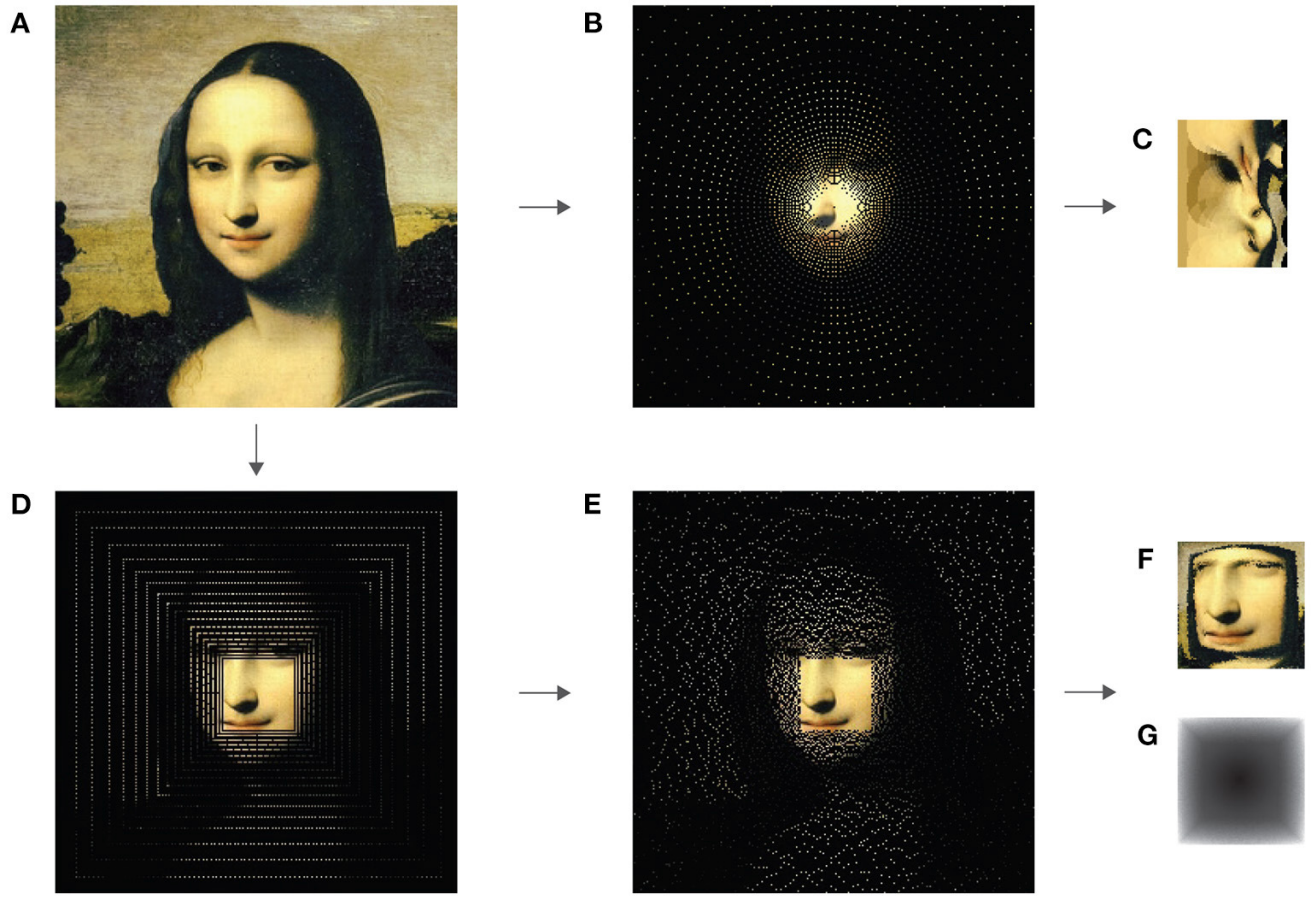
FCG foveation, used in our model (A → D → E → F). Log-polar transform (A → B → C) is provided for comparison. Notice that (B), (D) and (E) illustrate coordinates of the sampled pixels, represented by their true RGB value. The black background is meant to provide contrast for better visualization and is discarded during foveation. **(A)** Original image. **(B)** Log-Polar sampling. **(C)** Log-polar transform. **(D)** FCG sampling. **(E)** FCG sampling with jitter. **(F)** FCG foveated image. **(G)** A channel indicating sampled pixel positions.

A general scheme of the proposed model is shown in Figure 1. It consists of 3 main parts. First, the raw image input is sampled by the FCG sampling matrix (Figures 2D,E) with the starting position at the image center. Then, the foveated image is used as input to a standard image classification CNN architecture. The CNN produces two outputs—a classification prediction, used for error minimization at training, and the activation of the feature maps in the last convolutional layer, averaged on the features axis. The latter output we refer to as attention map *M*. Since locality and spatial relations between features are preserved in a CNN, we use the most salient features as an indication of a region of interest. Finally, the sampling matrix is translated to the most salient region on field of view and the process is repeated for the next time step. While we define the foveation and attention steps in more detail in the next two subsections, here we provide some details about how the neural network is constructed, trained and tested.

We use a standard classification CNN architecture with *softmax* output for class probabilities. There are a few restrictions on the CNN. We use batch normalization and Rectified Linear Unit (ReLU) activation function after convolutional layers in order to produce features that the attention mechanism can take advantage of. Both have been shown to speed up convergence and prevent exploding/vanishing gradient problem (Hochreiter, 1991) when training deep neural networks (Ioffe and Szegedy, 2015; Xu et al., 2015; Santurkar et al., 2018). For these reasons they are a standard component of almost all modern CNN architectures. Optionally, a Global Average Pooling layer can be used to extract features from the last convolutional layer. Although this component is not compulsory, it speeds up inference and has been shown to provide a regularization effect when used instead of a fully connected layer near the end of a CNN (Lin et al., 2013). In our model it also provides an easy and simple way of performing top-down attention by constructing Class Activation Maps (Zhou et al., 2016). All three components are standard and regularly used in modern CNN architectures. This combination allows our model to perform object recognition on the foveated image and provide means for bottom-up and top-down attention in a single forward pass of the network. Thus, the most salient object could be examined in detail (bottom-up) or a specific object could be tracked (top-down).

Our model has two variations depending on the signal being classified—a single image or a sequence of images (video input). On single images we perform the following procedure in both—training and testing phase. First, the image is foveated, with a centered sampling matrix, and processed by the CNN. Once we acquire the bottom-up attention map *M*, we translate the sampling matrix to the location on the raw image that is indicated as most salient. The image is foveated again with fovea placed in the suggested region and the result is processed by the CNN for a second time. We log both results in order to measure how classification improves after the fovea is centered at the region of interest. In order to adapt the network for video classification, we make a simple adjustment to the architecture by adding a LSTM recurrent layer (Hochreiter and Schmidhuber, 1997) before the softmax output. Training and testing is performed identically, with the exception that the sampling matrix is applied only once per frame. This model can take advantage of the sequential information of the video and compensate for cases in which the fovea is mistakenly positioned at a wrong place or the object of interest is occluded in one or more successive frames. Since the CNN processes foveated input, instead of full images, no pretrained networks could be used in the experiments. All models have been trained with gradient descent for at least 50 epochs with early stopping (Yao et al., 2007).

The CNN we use is based on the Xception architecture (Chollet, 2017), adapted to the size of the input and requirements of the model. For example, a global average pooling layer is used instead of a fully connected layer between the last convolutional layer and the softmax output. In order to avoid overfitting in versions of the model that train on smaller sized foveated images or a smaller dataset, we additionally decreased the number of weights in the network. All networks are trained with the original hyperparameters for at least 50 epochs with an early stopping mechanism. No fine-tuning and no pretrained weights were used in the experiments, since such weights are currently unavailable for foveated data.

### 3.2. Foveation

As a foveation mechanism, we use the FCG method, computed according to the algorithm in Table 2 in Mart́ınez and Robles (2006). The mapping is from coordinates in the raw image domain to coordinates of the foveated domain (*x, y*)⇒(*u, v*). We refer to this mapping as a sampling matrix (visualized in Figure 2D). The inverse mapping is given simply by a reverse of coordinate pairs (*u, v*)⇒(*x, y*). These mappings are computed only once. When foveating an image, pixel values are simply extracted at the relevant coordinates. In order to perform an “eye-movement” we translate the position of the sampling matrix by adding integer values Δ*x*∈ℤ and Δ*y*∈ℤ to the (*x, y*) coordinates in the image domain. Ideally, in an open-world environment, positioning the sensors outside of the current field of view will result in sampling some sensory information. As we test on 2-dimensional images or videos, though, we cannot rely on the same principles. Therefore, when the sampling matrix is displaced from the center of the image, some sampled values might lie outside its scope. In order to distinguish such cases from actual sensory input in the regular 0–255 range, we assign a value of –1.

It has been reported that the irregular sampling grid outside the foveal region in the retina prevents aliasing artifacts in vision (Yellott, 1983). In order to take advantage of such benefits, we introduce some jitter in the sampling coordinates of the peripheral rings of FCG (visualized in Figure 2E). While this irregular placement is beneficial for preventing aliasing distortions, from statistical sampling theory we know that it, unfortunately, also has negative effect on signal reconstruction. It has been shown, however, that a non-uniform sampling grid can still provide perfect reconstruction given the positions of sampling points are known (Strohmer, 1997). The human visual system does in fact possess mechanisms for determining cone positions (Hirsch and Miller, 1987; Ahumada Jr and Mulligan, 1990). In order to provide such information to our model, an additional channel δ(*x, y*) is added to the original image, as described in Wang and Veksler (2018), where the position of each pixel is indicated by the Euclidean distance between its coordinates and the center of the image. The image is then foveated using the jitter FCG sampling along all 4 channels. Foveation of the δ channel is visualized in Figure 2G. Based on the above-mentioned studies we test the effects of an irregular sampling grid in terms of classification accuracy in 4.

### 3.3. Attention

In order to guide eye-movements, a space variant model needs an attention mechanism. Here, we propose a mechanism for bottom-up and top-down attention with minimal requirements on the architecture and explain how eye-movements are guided and performed by the model. While most models train an attention mechanism separately from the main objective, here we present a method that exploits task-relevant features in the CNN in order to perform saccadic movements.

#### 3.3.1. Bottom-Up Attention

The task of a convolutional layer is to extract features from the previous layer and organize them in feature map units. Every filter that constructs a feature map is applied at each location of the given input—a property responsible for translation invariance in CNNs (Kayhan and Gemert, 2020). For this reason, features preserve their relative location throughout the network depth. Thus, by detecting the location of salient features we can localize potential regions of interest in a single forward pass. Salient features we define as features that contribute, or provide evidence, to the objective of the network. In our case this is classification, but the principle can be applied to other objectives as well, such as reconstruction, regression and so on. It is important to note that even if a set of features, e.g., in the periphery, does not lead to a correct classification, they still indicate a region of interest that can be explored by the fovea in a next time step, and therefore help improve classification over time. For example, if a fur texture is registered in the periphery that leads to the incorrect recognition of a dog, the agent can make a saccade to this location and correctly identify the source of the pattern as, e.g., a cat or a coat, instead.

In order to detect salient features, we use the mean of the last convolutional layer's activations across the features axis and receive a two-dimensional attention map *M* (visualized at the bottom of Figure 1). We use this map as an indicator of salient features and then direct the fovea toward the location of the highest intensity in *M*. This process is made possible by two principles in the neural architecture. First, ReLU activation functions are used in the network that pass positive values unchanged and map non-positive values to 0. This leads to the restriction of activations in the non-negative range where greater activations are more impactful on subsequent layers. The usage of max pooling layers in the network additionally contributes to this bias over high values associated with the propagation of informative features. Second, we use batch normalization after convolutional layers, similar to the saliency mechanism described in Itti et al. (1998). This layer normalizes separately the output of each feature map and thus eliminates amplitude differences between them. This allows us to construct *M* without giving priority to arbitrary filters over others in terms of intensity. Both, ReLU functions and batch normalization, are standard components of most modern CNNs and are therefore mostly not restrictive on the choice of architecture.

Now that we know high intensities in *M* indicate salient features, we need a mechanism that extracts a location for the next saccade. First, we map locations in the attention map *M*(*i, j*) to locations in the foveated image Φ(*u, v*); and then map locations from the foveated domain Φ(*u, v*) to the image domain *I*(*x, y*). As we use Log-Polar transform as a baseline sampling technique, for simplicity, we will use coordinates denoted by (*u, v*) for both mappings (FCG and Log-Polar), instead of the classical (ρ, θ) in Log-Polar:


(1) $$(u,v)=\left(\;log\left(\sqrt{{\left(x-x_{f}\right)}^{2}+{\left(y-y_{f}\right)}^{2}}\right),\;arctan\left(\frac{y-y_{f}}{x-x_{f}}\right)\;\right)$$

where *x*_*f*_ and *y*_*f*_ are coordinates of the focus point on the image, usually the image center. Since inverse mapping from the foveated domain to the image domain is known for both techniques, the attention mechanism is indifferent to how the image was foveated. We also define Φ_*inv*_ to be the inverse mapping function for coordinates (*u, v*)⇒(*x, y*) from the foveal domain to the image domain as described in Mart́ınez and Robles (2006) for FCG and in Qi et al. (2009) for Log-Polar transform. Using these notations, we calculate the most salient location (*u*_*max*_, *v*_*max*_) in Φ by scaling coordinates of the highest intensity location in *M* to Φ's dimensionality. Φ_*inv*_(*u*_*max*_, *v*_*max*_) then gives us coordinates (*x*_*max*_, *y*_*max*_) in the image domain, where a saccade is made in the next time step.

#### 3.3.2. Top-Down Attention

In order to implement top-down attention in our model, we use Class Activation Maps (CAM) (Zhou et al., 2016). CAM requires a Global Average Pooling (GAP) layer between the last convolutional layer *C*_*n*_ and the *softmax* output of the network. This layer is used in many standard CNN architectures and provides benefits to training, independent of its purpose in this model. However, this component only provides us with a convenient simplification for the purposes of this paper. Alternative methods like Grad-CAM (Selvaraju et al., 2017) or excitation backpropagation (Zhang et al., 2018) can be used with no modifications to the ANN architecture.

The GAP layer provides a direct interface to augmenting the attention map *M* such that it shows the contribution to each specific class' score. GAP pools feature maps *f*_*k*_ into *k* units (one for each), that are in turn connected to the *softmax* output through weights. If *f*_*k*_(*i, j*) denotes the output of each feature map in *C*_*n*_ at each spatial location, the class activation map for each classification output unit is given by:


(2) $$\begin{array}{r} M_{c}\left(i,j\right)=\underset{\begin{array}{c} k \end{array}}{\sum }w_{k}^{c}f_{k}\left(i,j\right) \end{array}$$

where $w_{k}^{c}$ is the weight corresponding to class *c* at unit *k*. Extracting the most probable location from *M*_*c*_ for class *c* is indicated by the highest intensity unit and can be extracted in the same manner as in the bottom-up approach. This way, even if features indicative of this class are not the most salient ones in the bottom-up direction, an agent can bias its eye-movement strategy in order to prioritize for a specific task. For example, when looking for a needle in a haystack, features of the needle, e.g., metallic color, will be prioritized over others.

## 4. Experimental Results

In order to validate the performance of the proposed model and examine its properties, we test on two different datasets. The first one is ImageNet (ILSVRC2010) (Russakovsky et al., 2015), containing images in 1,000 classes. We use this dataset in order to test for object recognition performance on single images. The second dataset-Core50 (Lomonaco and Maltoni, 2017), contains videos in 10 categories of 5 similar objects each, resulting in 50 classes. This dataset we use to more elaborately test the attention mechanism and how the model performs on video classification tasks. While images in ImageNet might suffer from photographer's bias, the objects in Core50 are smaller and appear at various positions in the image. As a preprocessing step, all raw images are scaled to 256 × 256 pixels with three color channels.

### 4.1. Computational Efficiency

**Figure 3:**
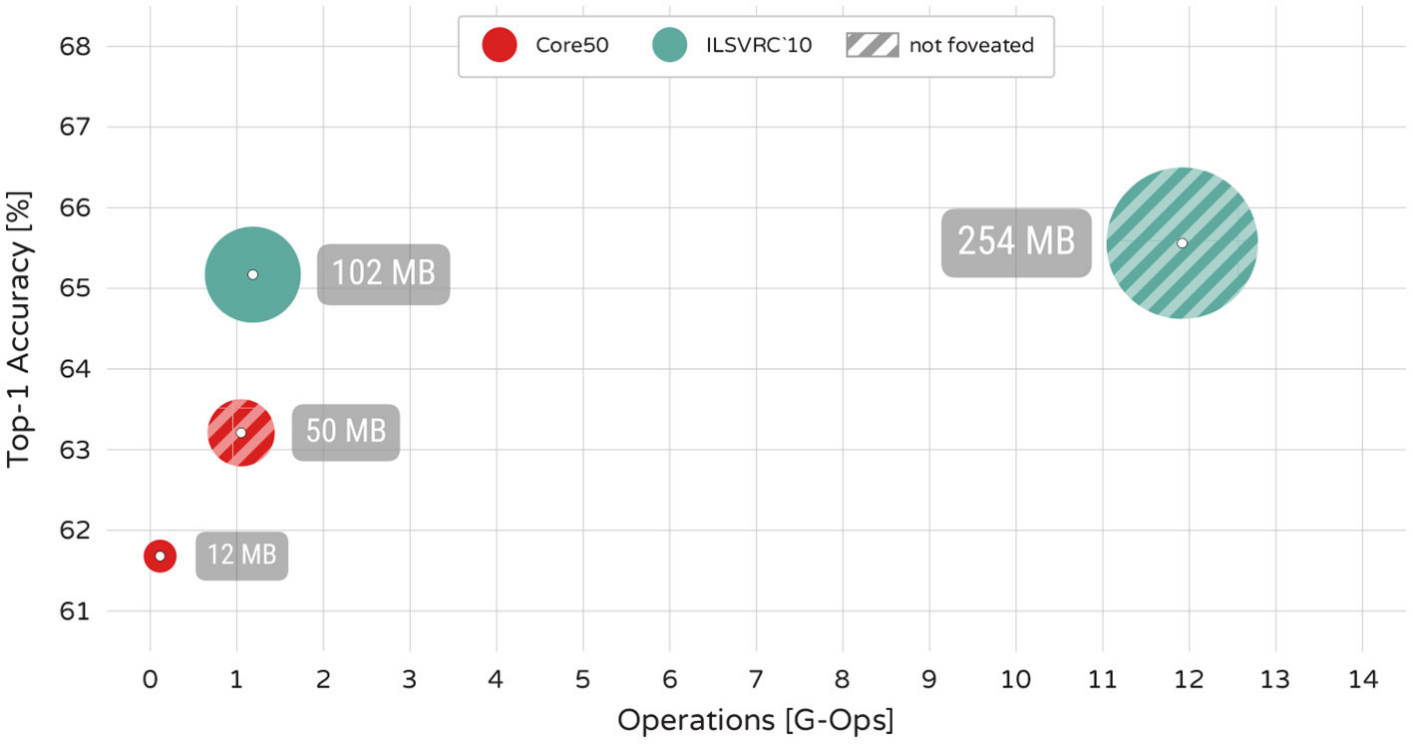
Computational cost of neural networks trained on foveated images (red and cyan) and full size images (shaded). The size of each circle indicates video memory consumption. Both, memory and floatpoint operations, are presented for a batch of 1 image.

A fundamental property of foveated vision is the reduction of sensory input and therefore computational costs required for processing the signal. Here we examine how the FCG sampling affects the size and computational costs of the neural network. We train two versions of our model for each dataset - one on full sized images, and one on FCG foveated images. The size of the foveated input is 81 × 81 pixels ≈10% of the pixels in the original 256 × 256 pixel images. The results and resource consumption of these networks are shown in Figure 3.

The foveal model trained on ImageNet is ≈10.06 times faster, uses ≈2.49 times less memory and has only 0.39% decrease in accuracy compared to the model trained on full size images. The foveal model trained on Core50 is ≈9.23 times faster, uses ≈4.16 times less memory and has a 1.53% decrease in accuracy. Using foveated images thus clearly reduces processing requirements of the architecture approximately 10-fold in terms of speed and 2.5–4.2 times in terms of memory consumption for the given foveation factor, at the price of small decrease in accuracy.

### 4.2. Classification Performance

Now that we confirmed that using foveated signal our model has a notable improvement in computational costs, we wanted to explore its performance on image and video classification tasks. First, we compare the effects of using FCG and Log-Polar transform under equivalent image reduction factor. We denote a model trained on 81 × 81 pixel FCG foveated images as FCG_10, considering 81 × 81 = 6,561 ≈ 10% of pixels of the raw image were sampled. Similarly, a model trained on Log-Polar foveated images of size 70 × 94 = 6,580 ≈ 10% of the original image pixels, we shall refer to as LogPolar_10. The training and testing procedure for both models is the same, as described in 3.1:

1. A raw image is foveated with a fovea placed in the center of the image;
2. The foveated image is passed through the CNN;
3. The source image is sampled for a second time with fovea moved to a new location, suggested by the bottom-up attention mechanism;
4. The new foveated image is passed through the CNN;
5. The second classification result is logged and used as performance metric.

**Figure 4:**
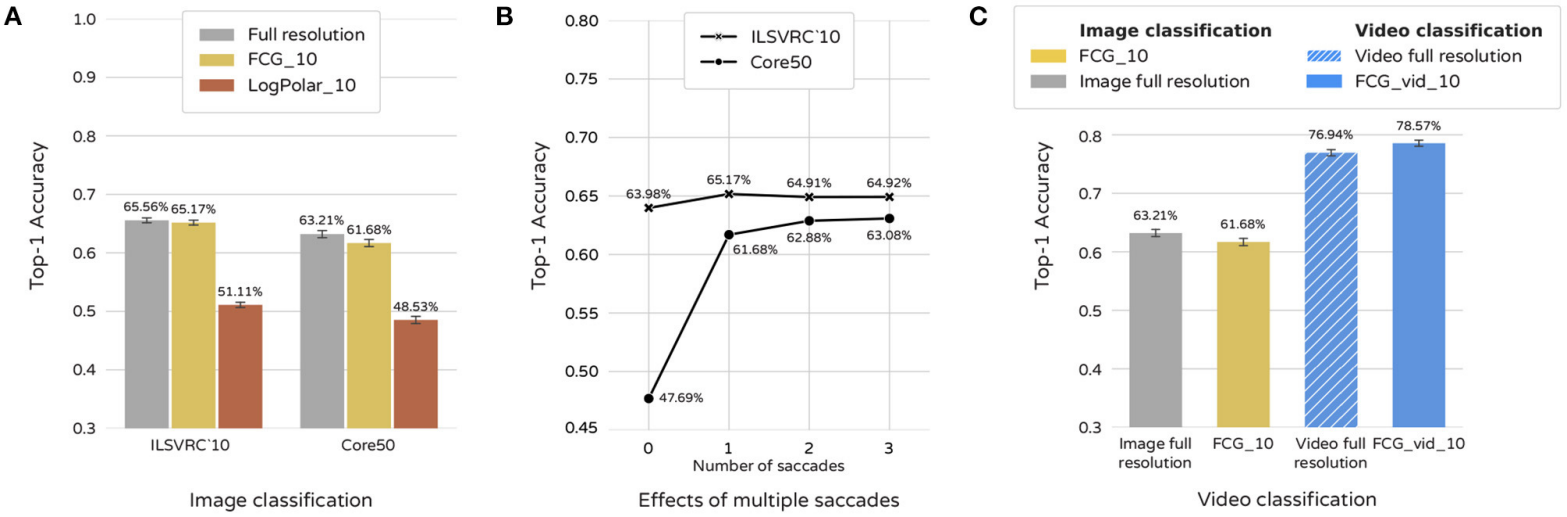
Classification performance. **(A)** Classification of FCG and Log-Polar foveated images when performing a single saccade. Results for full-resolution image classification are provided as a baseline. **(B)** Effects on performance of the FCG sampling model when multiple saccades are made per image. **(C)** FCG foveated videos and full resolution videos where sequential information is utilized by the model. The sampling matrix is only repositioned in each successive frame in FCG_vid_10 (i.e., a single sampling position per frame). Static image FCG_10 and full-resolution image classification are provided for comparison.

This way each model is given only one chance to locate a region of interest and view it in higher resolution. For the purposes of the test, we treat separate video frames in Core50 as standalone images. No sequential information is available to either model. Results of this experiment are shown in Figure 4A. FCG_10 outperforms LogPolar_10 by 14.06% on ImageNet and 13.15% on Core50.

While the model performs well on static images, foveation still leads to some loss of accuracy compared to full resolution images. Allowing the model to perform one or two additional saccades per image compensates for this effect as seen in Figure 4B. Effects on the ImageNet dataset are less prominent as one saccade is already enough to match full-image results. This might be due to most objects being centered and bigger in size.

Making additional saccades, however, comes at a computational cost. For this reason, we test our model on video classification, available only for the Core50 dataset. In this scenario, the model is allowed to process video frames in a sequence. Our intuition is that even though some information is inevitably lost during sampling, a short-term memory mechanism can improve performance by incremental collection of additional information through eye-movements. This can help correct for a failure of the periphery to accurately identify a region of interest, as well as other interfering factors, like object occlusions. We make a few changes to the training/testing procedure. First, a LSTM layer is added before the softmax output of the CNN. The resulting model we shall call FCG_vid_10. Second, the fovea is only centered on the first frame of a video. Its position in each successive frame is determined by the results from the previous one. As a result, the sampling matrix only has a single position per frame, opposed to classifying static images where the fovea is centered at first and then repositioned in each image. Video frames are used as input in sequences of 10, and the location of the fovea for subsequent frames is guided completely by bottom-up attention. The sequence length was chosen to maximize both models - trained on foveated data and full-resolution images. Further increase in the length of the sequence did not lead to improvements in performance. Results of this experiment can be seen in Figure 4C. While a model trained on full resolution videos has accuracy 76.94% (95% CI 76.41–77.47%), FCG_vid_10 scores 78.57% (95% CI 78.05–79.09%). Thus, although our model suffers a 1.53% decrease of accuracy in image classification, it outperforms full resolution input by 1.63% in video classification.

### 4.3. Sampling

**Figure 5:**
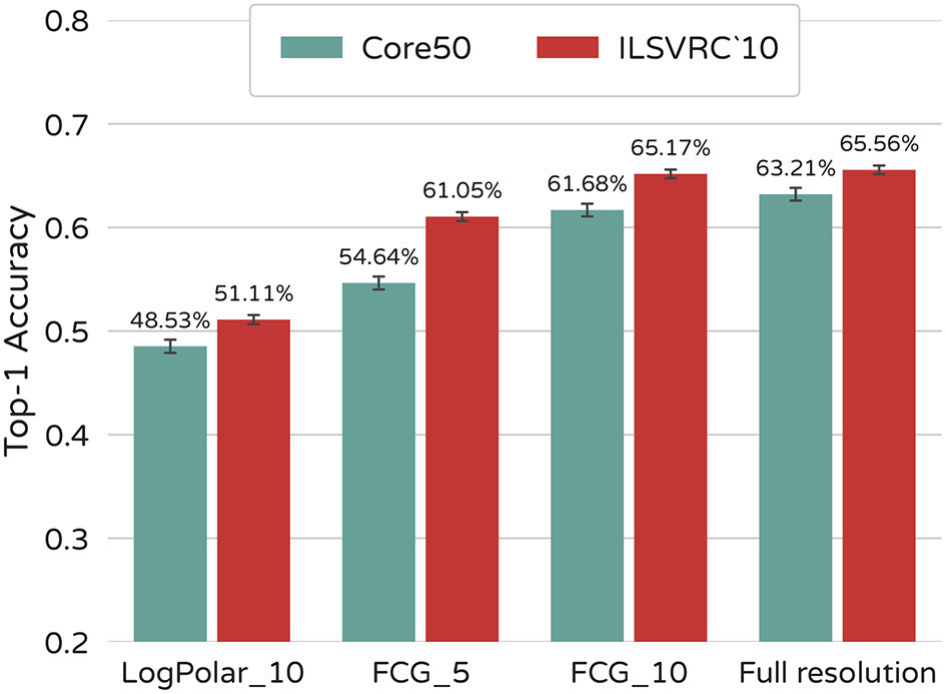
Effect of sampling sparsity on performance. FCG with 5% of pixels sampled, 10% sampled, and full resolution images. LogPolar_10 results are provided for comparison.

In order to test how the sampling sparsity affects performance of the model, we constructed a new FCG foveation mapping, where fovea and periphery are both reduced by half. The resulting foveal image is of size 59 × 59 = 3,364 pixels, which is ≈5% of the area of the source image. We call the model trained on the new foveated sampling rate FCG_5. Results are presented in Figure 5, as well as examples of the neural network input size. The decrease of accuracy is only 4.51% on ImageNet and 8.57% on Core50 despite the drastic decrease of pixels sampled from the source images. FCG_5 even outperforms the LogPolar_10 model by 6–10%. It is thus evident that FCG sampling scales sampling rate and network size to performance, demonstrating good flexibility of its usage.

In order to see how sampling affects classification, we performed tests with two types of sampling - classical FCG (Figure 2D) and with some jitter added to the coordinates of “photoreceptors” in the periphery (Figure 2E). The purpose of the latter one is to emulate the irregular sampling grid of the human eye. As non-uniform sampling is known to lead to difficulties for learning, it has been suggested that making the sampling point coordinates known to the algorithm can increase performance. We test this hypothesis in our model by adding an additional channel to the foveated image, indicating distance of each coordinate to the center of the image (depicted in Figure 2G). The methods we compared are: 1) FCG sampling, which is uniform in the fovea and locally uniform in the periphery (Figure 2D); 2) FCG + jitter-uniform in the fovea and non-uniform in the periphery (Figure 2E); 3) FCG + jitter + coordinates channel (Figures 2E,G); and 4) FCG+ coordinates channel (Figures 2D,G). As the results in Table 1 indicate, the approximating the irregular sampling grid of the retina leads to a small but not significant decrease in accuracy compared to basic FCG sampling. However, adding a coordinates channel does indeed significantly boost the performance of both types of sampling, while the greatest effects are on the locally uniform FCG sampling.

**Table 1:** Effects of jitter and information about sampling coordinates on the performance of the model.

**Method**	**ImageNet**		**Core50**	
	**Accuracy [%]**	**95% CI [%]**	**Accuracy [%]**	**95% CI [%]**
FCG_10	64.00	63.58-64.42	60.27	59.65-60.89
FCG_10 + jitter	63.31	62.89-63.73	60.18	59.56-60.80
FCG_10 + jitter + coord	64.09	63.67-64.51	60.66	60.04-61.28
FCG_10 + coord	**65.17**	64.75-65.59	**61.68**	61.06-62.30

*The bold value indicates highest accuracy method for each dataset*.

### 4.4. Attention

**Figure 6:**
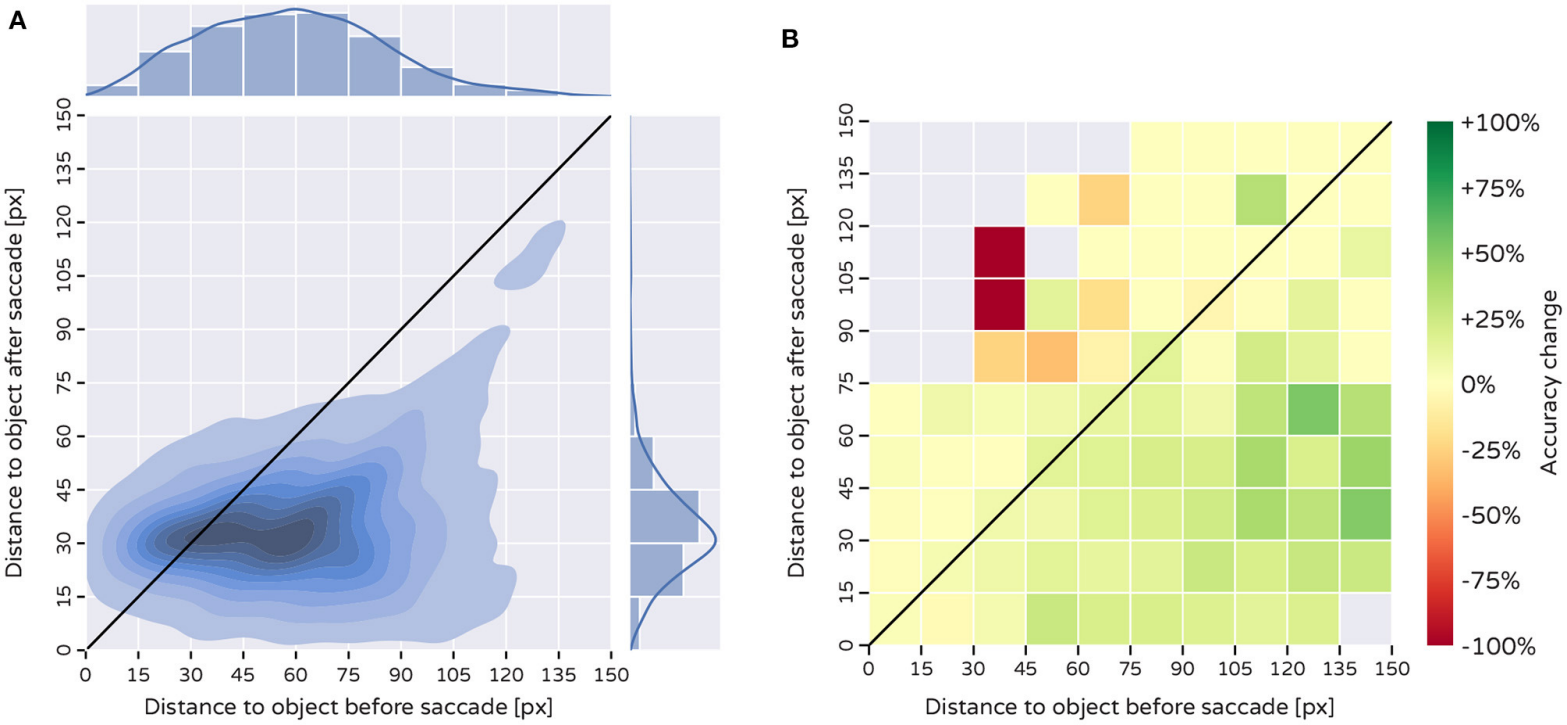
Effects of a single saccade on the displacement of the fovea from classified objects and the resulting change in accuracy. The fovea starting position is always in the center of the image. **(A)** Kernel density estimate of the euclidean distance between the centers of classified objects and the fovea center before and after a saccade. Distributions of the distances are shown for before (top) and after the saccade (right). **(B)** Change in classification accuracy resulting from the saccade. Accuracy change is calculated by subtracting the average accuracy of all samples that fall within a grid cell before a saccade was made, from the average accuracy in this cell after a saccade.

To further investigate the detection properties of the periphery, we study how the model uses the attention mechanism to make saccadic movements. We use the Core50 dataset, as positions of objects there are known, and they vary more than in ImageNet. We perform image classification on every frame in the test set and record how the distance of the center of the fovea changes in relation to the center of the object displayed. In order to perform a saccade, the fovea is first centered on the image and the resulting foveation is used to determine the next position of the sampling matrix. As the source images are of size 256 × 256 px, most objects are located in a 128-pixel radius from the center, except for a few that are displayed in the corners of the image. The largest possible distance is, at most ≈181 px-half the length of the diagonal. Using our experimental paradigm, we find that the attention mechanism draws the fovea closer to the classified objects in the majority of the cases (Figure 6A). After an “eye-movement” 96% of the object centers are located within 60 px, and 75% of the object centers are closer than 42 px to the fovea center. As a result, the initial distribution (Figure 6A top) is squished dramatically with a mean around the 30px bin (Figure 6A right). We find that accuracy increases with 12.13% after one saccade, where accuracy improvement is a function of the distance shortened after the saccade (Figure 6B). This provides evidence that even if an object is misclassified at the starting position of the sampling matrix, the majority of objects were correctly located within a region of interest, and accuracy improved once the high-resolution fovea moved closer. Thus, the efficiency of the bottom-up attention mechanism for performing “eye-movements” is evident.

**Figure 7:**
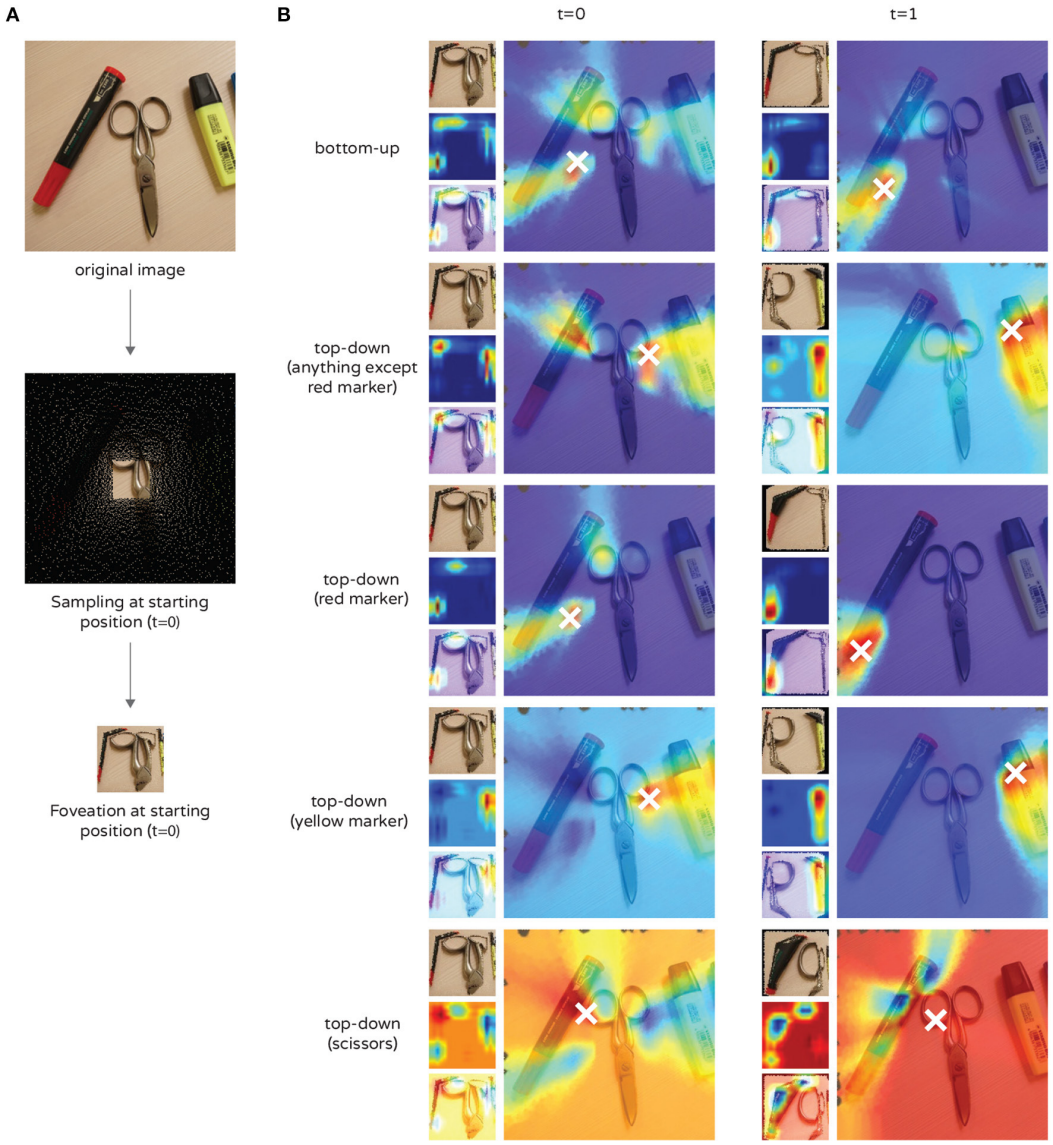
An example of the top-down attention mechanism. **(A)** Preprocessing steps - original image (top), positioning of the sampling matrix (middle) and the resulting foveated image used as input to the model (bottom). The black background (middle image) is meant to provide contrast for better visualization and is discarded during foveation. **(B)** Effects of attention (rows) on saccadic movements (columns). Each cell contains 4 images: foveated image at the current foveal position, used as model input (left-top); the resulting attention map, upsampled for improved viewing (left-middle); attention map superimposed on the model input (left-bottom); and the attention map superimposed on the source image (right). A white “x” indicates the location of the highest intensity pixel in the attention map, i.e., location of the next saccade.

In addition to the bottom-up attention, we demonstrate the application of Class Activation Maps in the top-down direction for our model (Figures 7A,B). The figure shows an example of a cluttered scene with 3 objects—a red marker, scissors and a yellow marker. In each row of Figure 7B, the sampling matrix is centered, and one saccade is made. The attention map of the model was upsampled for better viewing and an inverse mapping to the source image domain was made (resembling Figure 2E). The result was then interpolated for better visualization and superimposed on the source image, visualizing an attention heatmap. The first row of the figure shows the bottom-up direction, where the red marker appears to have the most salient features. Therefore, a saccade is made in its direction (row 1, column 2). We denote the bottom-up attention map *M* and class activation maps *M*_*r*_, *M*_*y*_, and *M*_*s*_ for the classes of the red marker, yellow marker and scissors, respectively, constructed as described in 3.3.2. The bottom three rows in the figure show a top-down attention bias by using only *M*_*r*_, *M*_*y*_, and *M*_*s*_. The attention mechanism then guides the fovea to three different locations on the image, correctly locating the desired objects. In row 2, we demonstrate inhibition of attention to a specific stimulus. This is done by acquiring a top-down map *M*_*custom*_ = *M*−*M*_*r*_, which suppresses any features of the red marker and indicates the yellow marker as the second most salient stimuli in the photo. This arithmetic can be done for conjunctions of different class activation maps in order to place or withdraw importance depending on the current task of the agent. Once an object is correctly located and classified, the agent can therefore suppress its location in the activation map, or even inhibit the whole class. Thus, we demonstrate the ability of the agent to explore its environment incrementally or search for a specific, desired object in a cluttered scene, even if this object is not salient in the bottom-up direction.

## 5. Discussion

In this work we presented an end-to-end neural vision model of the ventral visual stream, inspired by the sampling mechanisms of the primate retina. We showed that by foveating the visual signal it performs comparably in accuracy for image classification and outperforms models with full-resolution input in video classification. At the same time, there is a dramatic decrease in computational costs, as speed is increased 10-fold and memory required is reduced several times. Additionally, we show empirically that Foveal Cartesian Geometry outperforms Log-Polar transform foveation on classification tasks and under similar foveation constraints. We demonstrated an easy to implement, inbuilt attention mechanism based on feature saliency in the bottom-up direction and show how top-down attention can be utilized for incremental exploration and goal-driven search.

One issue of the proposed model is that the foveation mechanism is not a viable option for embodied robotics in the current state. FCG can be used in virtual environments or simulations for training agents. In order to use FCG in robotics, however, a high-resolution image needs to be obtained first, which is undesirable since most of it will be discarded anyway. However, there are some hardware implementations which produce very similar results and can be used instead of FCG. A more closely related implementation is an array of cameras, like in Carles et al. (2016), that can be arranged to reproduce a similar foveation effect. Another solution is using a camera lens (Kuniyoshi et al., 1995; Martinez et al., 2001), inspired by the human eye that can project the signal to a classic CCD or CMOS chip. A broader overview of other hardware implementations and patents of sensors for foveal vision is presented in Weber and Triesch (2009).

Another topic which has not been discussed so far in this work is the usage of a classification CNN as the basis of the model. The class outputs are not necessary for the bottom-up attention mechanism, but they are crucial for the top-down mechanism to function. However, different objectives are equally plausible for the vision model, as long as there is a semantically separable representation that can be linked to attention. For example, a β-VAE (Higgins et al., 2017) model can be used, instead. This neural network is an autoencoder that learns interpretable factorized representations of independent data generative factors. The hidden units are not classes, but independent factors learned in an unsupervised manner. They have the property of being semantically interpretable and can serve the same purpose as the class output units in our model. Instead of classes like “cat” or “dog,” they usually encode features like pose, shape, illumination and other independent factors of the observed data. Furthermore, it has been suggested that ventral stream representations in the primate brain encode very similar factors likely to be obtained through unsupervised learning instead of categorization (Christensen and Zylberberg, 2020). In this sense, further future efforts are needed in order to extend our work to a more general-purpose vision model.

The irregular grid of photoreceptors outside the human fovea is theorized to reduce aliasing artifacts in vision at the cost of vision impediment (Yellott, 1983). Results from literature, however, show that such reduction is not observed in humans and could be compensated by keeping information about photoreceptor locations on the grid (Strohmer, 1997). Our results confirm that irregular sampling leads to small reduction of performance, which can be in fact compensated by providing information about sampling positions to the neural network. This finding shows that performance of the semi-irregular sampling in FCG can be enhanced by supplying an additional channel to the visual input. Furthermore, we showed that even more intense jitter, similar to the one observed in biological systems, would not affect vision crucially, which on one hand confirms biological findings, and on the other—makes requirements on building a sampling matrix less rigid. In effect, such sampling could be collected by a multitude of crudely assembled sensors in future developments of artificial retinas, compatible with self organized assembly seen in biological systems.

In recent years foveation gained a lot of attention from research in the fields of signal compression and reconstruction. Some clear directions are for Virtual Reality rendering (Guenter et al., 2012; Patney et al., 2016; Hsu et al., 2017), video streaming (Lee et al., 2001; Lee and Bovik, 2003) and gaming (Illahi et al., 2017, 2020). However, few vision models have been proposed that use foveation. Deep convolutional networks are currently the state-of-the-art methods for vision models. Even when carefully constructed, models like (Wallace et al., 1994; Colombo et al., 1996; Aboudib et al., 2015, 2016) that do not use CNNs can rarely compete in terms of performance. Aboudib et al. (2016) for example follows very similar principles to our model, despite the lack of a top-down mechanism. The use of log-polar sampling they use prevents the deployment of CNNs and allows only for shallow machine learning algorithms.

Daucé et al. (2020) make a compromise, instead. They feed a foveal crop to a CNN and use log-polar sampling with a shallow ANN as an attention mechanism, emulating a dorsal stream. This method, however, is hard to train, as correct foveal classification is necessary as a metric for training the attention mechanism, but a functional attention mechanism is needed in order to train the foveal network in turn. Thus, the model has to be trained offline with a reinforcement learning technique. Another model (Jaramillo-Avila et al., 2019) implements a very similar to FCG foveation technique and deploys bottom-up and top-down attention mechanisms for object detection in mobile robots. They use a CNN to process the foveated image but the attention mechanism they use is based purely on saliency heuristics and is not learned. It also requires a full resolution image and uses several image pyramids in order to compute the bottom-up saliency map.

As our model is primarily focused on the ventral “What” pathway, a natural next step would be to extend the model with a dorsal “Where” pathway. Rod cells, for example, contribute mostly to the dorsal stream. Although they are believed to saturate under photopic conditions, it has recently been discovered that they escape saturation and participate in day light vision in mice (Tikidji-Hamburyan et al., 2017). As it is very hard to separate the signal of rod cells, this matter is challenging to study. It is currently not known if this is also the case for humans. Regardless of biological plausibility, however, such an addition to a vision model might present some advantages for better spatial attention in the periphery. Rod cells are much less foveated and their signal could provide a coarse-grained low-resolution information that compensates for distortions caused by foveation. Additionally, spatial awareness of the agent can be improved with a more elaborate short-term memory mechanism and motion sensitivity—another feature usually associated with the dorsal pathway.

## 6. Conclusion

In this paper we proposed a new deep learning vision model inspired by the structural organization of the primate retina. The information reduction provided by the foveation technique allows for an energy efficient way to perceive a visual scene and acquire detailed information incrementally. Parts of the observed scene can be explored with high resolution, while the agent retains a large field of view in low resolution. Moreover, we provided an easy to implement attention mechanism that allows for saccadic “eye movements” to be performed in order to acquire more detailed information from different parts of the scene in a sequential manner. We believe this shift in paradigm from classical machine vision can assist development of embodied active vision agents in simulated environments or in the field of cognitive robotics.

## Data Availability Statement

Publicly available datasets were analyzed in this study. This data can be found at: 1. “ImageNet Large Scale Visual Recognition Challenge” at “https://image-net.org/challenges/LSVRC/2010/2010-downloads.php”; 2. “CORe50” at “https://vlomonaco.github.io/core50/”; Source code available at https://github.com/hlukanov/fovperi.

## Author Contributions

HL designed and performed the experiments, derived the models, analyzed the results, and wrote the manuscript. GP and PK supervised the project and guided the writing process. All authors contributed to manuscript revision, read, and approved the submitted version.

## Funding

The project was financed by the funds of a research training group “Computational Cognition” (GRK2340) provided by the Deutsche Forschungsgemeinschaft (DFG), Germany.

## Conflict of Interest

The authors declare that the research was conducted in the absence of any commercial or financial relationships that could be construed as a potential conflict of interest.

## Publisher's Note

All claims expressed in this article are solely those of the authors and do not necessarily represent those of their affiliated organizations, or those of the publisher, the editors and the reviewers. Any product that may be evaluated in this article, or claim that may be made by its manufacturer, is not guaranteed or endorsed by the publisher.
